# Weeds and ground-dwelling predators′ response to two different weed management systems in glyphosate-tolerant cotton: A farm-scale study

**DOI:** 10.1371/journal.pone.0191408

**Published:** 2018-01-19

**Authors:** Esteban García-Ruiz, Íñigo Loureiro, Gema P. Farinós, Pablo Gómez, Elena Gutiérrez, Francisco Javier Sánchez, María Concepción Escorial, Félix Ortego, María Cristina Chueca, Pedro Castañera

**Affiliations:** 1 Instituto Nacional de Investigación y Tecnología Agraria y Alimentaria (INIA), Departamento de Protección Vegetal, Laboratorio de Malherbología, Madrid, Spain; 2 Centro de Investigaciones Biológicas, CSIC, Departamento de Biología Medioambiental, Laboratorio de Interacción Planta–Insecto, Madrid, Spain; Georg-August-Universitat Gottingen, GERMANY

## Abstract

The use of glyphosate, as a post-emergence broad-spectrum herbicide in genetically modified glyphosate-tolerant (GT) cotton, supposes a big change in weed management programs with respect to a conventional regime. Thus, alterations in arable flora and arthropod fauna must be considered when evaluating their potential impacts. A 3-year farm-scale study was conducted in a 2-ha GT cotton crop, in southern Spain, to compare the effects of conventional and glyphosate herbicide regimes on weed abundance and diversity and their consequences for ground-dwelling predators. Surveys reveal that weed density was relatively low within all treatments with a few dominant species, with significantly higher weed densities and modifications of the floristic composition in glyphosate-treated plots that led to an increase in the abundance of *Portulaca oleracea* and to a reduction in plant diversity. The activity-density of the main predatory arthropod taxa (spiders, ground beetles, rove beetles and earwigs) varied among years, but no significant differences were obtained between conventional and glyphosate herbicide regimes. However, significant differences between treatments were obtained for ground beetles species richness and diversity, being higher under the glyphosate herbicide regime, and a positive correlation with weed density could be established for both parameters. The implications of these findings to weed control in GT cotton are discussed.

## Introduction

Herbicide tolerance has been the most prevalent genetically modified trait adopted by farmers since transgenic crops were first commercialized in 1996. Alone or stacked with other traits, genetically modified herbicide tolerant (GMHT) soybean, maize, canola, cotton, sugar beet and alfalfa have been deployed in developed and developing countries, reaching about 150 million hectares worldwide in 2015 [1]. GMHT crops allow an easier, cheaper and more efficient weed control, with simplified herbicide applications and a reduction in the risk of failed weed control arising from the post-emergence spraying of a non-selective and non-residual herbicide that, while facilitating crop rotations, also leads to a potential decrease in the environmental impact of the treatments [2,3]. However, the expansion of GMHT crops associated with a massive use of only one non-selective herbicide replacing the majority of the conventional herbicides used previously, without care in integrated weed management, may modify the composition and abundance of weed populations, allowing the prevalence of one or few species due to herbicide resistance or weed shifts, and adversely impact the agro-environment [4,5].

Weeds play an important role in agroecosystems supporting biological diversity within cultivated lands [6–8] and providing valuable ecological services (food, cover or reproductive sites) for arthropods and their natural enemies [9–11]. Thus, trophic relationships must be considered when evaluating how new technologies such as GMHT crops affect weed management. No direct detrimental effects of GMHT crops on pest and beneficial arthropods have been reported yet [12–14]. Although most studies show no acute or chronic effects of glyphosate-based herbicides on terrestrial arthropods [15,16], negative effects have recently been cited on non-target invertebrate populations such as aphids or earthworms [17,18]. Some studies indicated that glyphosate may interfere with chemical communication and prey capture behavior of some predators [19–21]. The different use of herbicides in GMHT crops could alter the species composition of predatory communities by changing the vegetation diversity and the biomass available [14,16]. Concerns have been expressed that if the GMHT strategy becomes widely adopted, weed flora and arthropod abundance and diversity could be reduced in the long-term [22], exacerbating the current trends of continuous decline of farmland biodiversity by intensive agriculture systems [23,24]. The Farm Scale Evaluation of GMHT beet, oilseed rape and maize conducted in the United Kingdom showed that the abundance of most arthropod taxa were highly influenced by season and crop species, and by GMHT management to a lower extent, following the effects herbicides on the abundance of their resources [25]. In general, herbivores, pollinators and natural enemies abundance was smaller in GMHT beet and spring oilseed rape but larger in GMHT maize, whereas detritivores increased in abundance under GMHT management across all crops [26,27]. Multiyear field-trials have also been performed for GMHT soybean in Iowa [28], GMHT beet in Denmark [22] and GMHT maize in Spain [29,30], Hungary [31] and Czech Republic [11]. In general, few negative effects on beneficial insects were found, the effects of GMHT cropping on arthropod populations depending on the timing of herbicide application and the different efficacies of weed-control regimes compared with conventional crops.

Cotton (*Gossypium hirsutum* L.) is one of the most important non-food crops in many regions of the world and one of the most important socioeconomic crops in southern Spain. Its products are destined to different industries as textiles, food or chemicals. Herbicide-tolerant cotton cultivars were commercially available for the first time in 1997 in the United States, accounting for 89% of the cotton hectares planted in the USA in 2016 [32]. Currently there are no GMHT crops authorized for cultivation in the European Union (EU), but several crops including cotton have been approved for food and feed uses, import and processing.

In order to minimize yield losses, weed control is essential during the cultivation period of cotton. Conventionally, pre-emergence chemical control of weeds using residual herbicides allows the adequate establishment of the cotton plants that will be able to compete successfully with developing weeds. But the introduction of only glyphosate treatments in post-emergence associated with continuous glyphosate-tolerant (GT) cotton cropping has led to shifts in weed species composition [33]. Two surveys conducted in Georgia (USA) in 1995 and 2005 on farmers′ opinions about weed control in GT cotton with glyphosate resulted in five of the ten most troublesome species in cotton were common to both surveys while the other five, susceptible to glyphosate in 1995, were replaced in 2005 by other species of the genera *Amaranthus*, *Commelina*, *Ipomoea*, *Richardia* or *Cyperus*, which were not effectively controlled by glyphosate. Of them, *Commelina benghalensis* is tolerant and *Amaranthus palmeri* resistant to glyphosate [33,34]. These changes in the vegetation could have implications on the species composition of predatory communities. A two-year farm-scale evaluation of commercial fields in Arizona showed that cultivation of both non-transgenic cotton and GMHT cotton stacked with *Bt* resulted in a decline of ants and an increase of beetles, when compared with adjacent non-cultivated sites, but no differences were observed between both types of cotton [35]. In addition, a study comparing weedy and weedy free cotton showed that the presence of weeds was associated with greater populations of natural enemy arthropod groups [36]. Nevertheless, there is little information on the interactions between different weed management practices and the abundance and diversity of natural enemies in GT cotton, although this information is essential for developing biologically based Integrated Pest Management programs.

To our knowledge, this is the first study performed in the EU in cotton to investigate the relationship between weed management and the beneficial predatory arthropods in a GT cotton crop. The objective of this long-term field-scale study is to provide insight into the impact of GT cotton crop subjected to two different weed management regimes on weed and ground-dwelling predatory communities. Specifically, we compare a conventional regime consisting on pre- and post-emergence herbicide applications, according to the recommended regional practices for cotton production; and another one based on glyphosate only application in post emergence. For this aim, changes in the density, species richness, diversity and seasonal phenology of plants and predator communities under both herbicide regimes have been assessed in a three-year farm-scale study in southern Spain.

## Materials and methods

### Site description

The experimental site was a commercial cotton field located at Lebrija, in the province of Sevilla, Andalucía, southern Spain (36°59′ N, 6°5′ W, 1 m.a.s.l.). This region is one of the traditional cotton producing areas, where is grown 98% of existing cotton in Spain [37]. The field was cultivated with cotton for at least the two previous years and populations of weeds naturally occur in it. The soil at the site of study was of clayey nature with low sand content, alkaline and with low organic matter content [38].

This site is under a typically Mediterranean climate, with mild and wet winters and autumns and long dry summers. The 30-year average annual rainfall is of 598 mm, mostly distributed from October to May and almost absent during the summer. Rainfall during the growing seasons (May-August) was 43, 9 and 39 mm in 2008, 2009 and 2010, respectively, while 30-year average for the corresponding period is 58 mm. Climatic data were obtained from “Lebrija I” weather station (36°58’40”N, 6°7’30”W, 25 m.a.s.l.).

### Experimental design

The experiments were carried out within a 2-ha field sown with “Glytol” glyphosate-tolerant cotton event ‘GHB614’ (Bayer CropScience) during the cotton growing seasons from 2008 to 2010. The trial was laid out as a randomized complete block design, with two treatments (weed-management regimes) and four blocks of two plots. The two treatments were randomly assigned to the two plots within each block and maintained for three years. Plot size was 60 m by 30 m. S1 Fig shows additional information on experimental design.

Each year, the field was chisel-ploughed and then disked and field cultivated for seedbed preparation. Then cotton was sown in a weed-free and finely prepared seedbed at a depth of 3 cm using a precision seeder at 150,000 seeds ha^-1^ (20 kg ha^-1^) in rows spaced at 95 cm. Cotton seed was supplied coated with the insecticide ‘Gaucho’ (Imidacloprid). The insecticide Teflutrin (0.5% w/w) was also applied at sowing at 8 kg ha^-1^ as soil disinfectant for early-season insect control, a common practice in conventional cotton production. No other additional insecticides were applied in the site since the initiation of the study. Sowing dates for this study were 7th July 2008, 12th May 2009 and 19th May 2010. In 2008, the sowing date was postponed until the beginning of July because of the delay in the availability of GM cotton seed.

**Fig 1:**
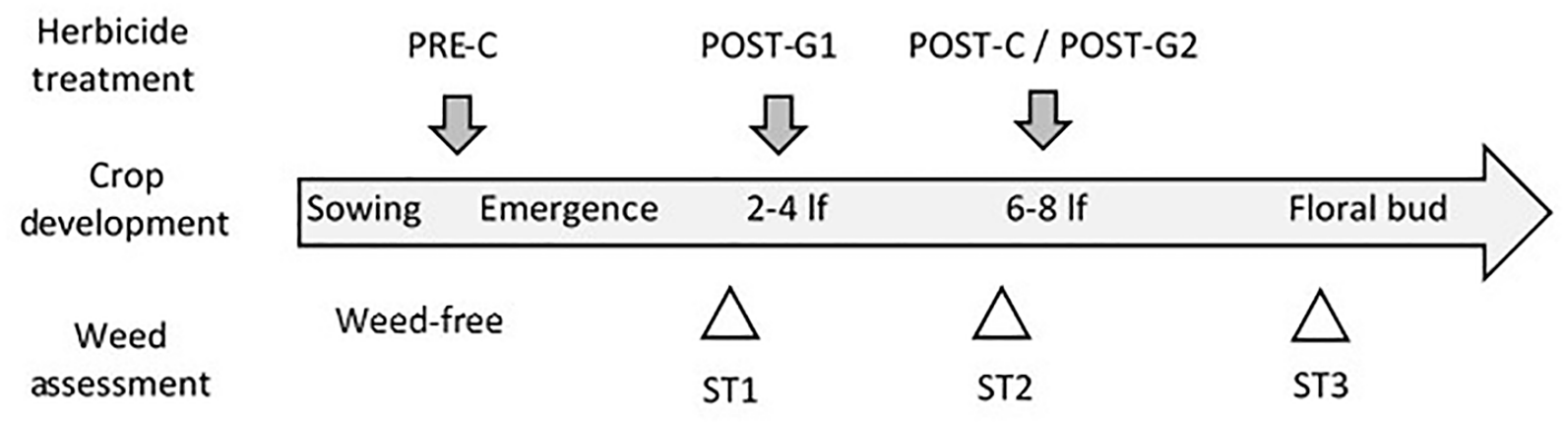
Scheme of the herbicide treatments and weed assessments conducted in genetically modified herbicide tolerant cotton. All plots were weed-free at cotton sowing. PRE-C and POST-C emergence herbicides were applied only in conventionally (C) treated plots. POST-G1 and POST-G2 (glyphosate treatments) were applied only in glyphosate treated plots (G). Weed assessment was conducted in both C and G plots at three times: ST1) a few days before the application of POST-G1; ST2) 2–3 weeks after POST-G1; and ST3) 2–3 weeks after POST-C/POST-G2.

Herbicide regimes consisted of: glyphosate-only (G) treatment and conventional standard (C) treatment, which was used as control (Table 1 and Fig 1). The G treatment included two applications of glyphosate in post-emergence (POST-G1 and POST-G2). The C program included a pre-emergence (PRE-C) herbicide application immediately after cotton was planted and a post-emergence (POST-C) application. The PRE-C herbicide possess residual activity has a broad-spectrum control of annual broadleaf and some grass weeds in their early stages of development. The POST-C graminicide was applied for annual grass weed control. In 2008, and because of the delayed sowing, there were no weeds in the conventional plots at the time of the post-emergence application, so POST-C treatment was not applied. All treatments were applied using a tractor-mounted sprayer with flat-fan nozzles calibrated to spray 180 L of solution per hectare.

**Table 1:** Herbicide regimes (active ingredient, dose and application time) used in a genetically modified herbicide tolerant cotton.

Herbicide Treatment	Active ingredient (Commercial product)	Timing^a^	Dosage (kg a.i ha^-1^)	Application date (plant stage)^b^		
				2008	2009	2010
**Conventional (C)**	Fluometuron 25% + Terbutilazine 20.8% (Cottonex NeoPro)	PRE-C	0.88 + 0.73	July 8	May 13	May 20
	Clethodim 12% (Centurion Plus)	POST-C	0.18	na^#^	July 1 (8 lf)	June 28 (6 lf)
**Glyphosate (G)**	Glyphosate 36% (Roundup)	POST-G1	1.08	July 31 (4 lf)	June 16 (4 lf)	June 15 (3 lf)
	Glyphosate 36% (Roundup)	POST-G2	1.08	August 20 (8 lf)	July 2 (8 lf)	June 29 (6 lf)

^a^ PRE: pre-emergence application (immediately after cotton was planted); POST: post-emergence application.

^b^ Plant stage: cotton in 3rd (3 lf), 4th (4 lf), 6th (6 lf), and 8th leaf stage (8 lf).

^#^ Herbicide not applied.

For a successful crop growth cotton, the same local agronomic practices, including tillage, were used for both weed management regimes. Cotton was irrigated by using an overhead sprinkler system every 2–3 weeks. The first irrigation was applied after sowing to ensure uniform germination, while next ones were applied always several days after each POST- herbicide application. The field was fertilized twice, with a basic dressing at a rate of 350 kg ha^-1^ NPK 7-10-6 before sowing and 350 kg ha^-1^ NPK 25-0-0 as top dressing. The conventional herbicide treatment was used to manage the weeds surrounding the experimental plots.

Due to the fact that GM cotton (not approved for commercial use in the EU) was grown in an intensive cotton producing area, and that cross-pollination of neighbouring commercial non-GM cotton fields would result in GM-contaminated cotton seed, all the cotton plants were hand extracted just before reaching the flowering stage each year (5th September in 2008 and 29–31 July in 2009 and 2010). This GM plant material was destroyed in a nearby field by incorporating it into the soil with a disk harrow in compliance with Spanish regulations. Weeds remained in the experimental plots until the end of their cycle.

### Weed assessments

Weed assessment was conducted in both C and G plots three times; ST1: a few days before the application of POST-G1; ST2: 2–3 weeks after POST-G1; and ST3: 2–3 weeks after POST-G2/POST-C (Fig 1). This time frame was chosen to get the best measure of the agronomic management impact on weeds, and to evaluate only those weeds that competed with the crop and would produce seeds for the next cycle.

Weed sampling was performed using 0.25 m^2^ quadrats. Counts of individual weed plants identified to species (abundance) were made in thirty-four quadrats per plot (a total area of 8.5 m^2^). To estimate weed density (plants m^-2^) and frequency, the quadrats were placed in a zig-zag pattern along two diagonal transects (ten quadrats per transect) and in the four borders (three quadrats per short border and four per long border) of each plot, leaving a buffer of three meters to avoid edge effects. Frequency (*Fi*) was calculated as the percentage of quadrats in each plot in which the species *i* was present.

### Arthropod assessments

Aboveground arthropods were monitored by using pitfall traps. Three traps were arranged diagonally across each plot, starting at least 10 m from the plot boundary to minimize potential edge effects. Each trap consisted of a plastic cup 12.5 cm in diameter and 12 cm deep with a plastic funnel fitted to the top, and both flush with the ground surface. An inner plastic 150 ml container half filled with a 1:1 mixture of water and ethanol was placed inside the plastic cup. The outer cup was punched to let the irrigation or rain water drain off. Traps were operative for 2 days every two weeks. A total of six sampling dates per year were performed from the end of May to mid-August in 2009 and 2010 and from the beginning of July to the end of September in 2008. All the individuals collected in the pitfall traps were taxonomically identified to the genus/species level in the main predatory groups and to at least the family level in the others. The number of arthropods captured in pitfall traps is a function not only of the density, but also of the activity of the sampled organisms and their behaviour on encountering a trap. Hence, the term ‘‘activity–density” will be used to refer to population estimates obtained using pitfall traps.

### Richness and diversity estimates

Species richness (*S*) and the Shannon-Wiener diversity index (*H*´ = -∑ *pi* ln *pi*, where *pi* is the relative abundance of each species) were used to detect changes in the community structure between herbicide management regimes for weed species and for the prevalent ground-dwelling insect predators collected (spiders, ground beetles and rove beetles).

### Statistical analysis

The effects of herbicide management on weed density were analysed by a mixed-model ANOVA (year, herbicide management and sampling time of weeds as fixed factors and block as random). Differences in arthropods activity-density were tested by a mixed-model ANOVA (year and treatment as fixed factors and block as random) with repeated measures (sampling date). All interactions were included in the models. Weed density (counts for all quadrats within a plot were pooled) data were log (x+0.5) transformed while arthropods activity-density (counts per pitfall trap) data were square root (x+0.5) transformed to stabilize variances. Weed and arthropod species richness and diversity data were pooled across sampling dates within plots and tested by two-way ANOVA (year and treatment) analyses. Pairwise comparisons were made by using the Student Newman-Keuls test (SNK) and the significance level of *P* < 0.05 was considered for all tests. All the analyses were carried out using the general linear model (GLM) procedure of IBM SPSS Statistics version 23.

Pearson’s bivariate correlation analysis was performed to investigate relationships between annual arthropod variables (spider, ground beetle and rove beetle activity-density, richness and diversity and earwig activity-density) and total weed density across plots. Tests of statistical bilateral significance for the matrix of correlations (a total of 30 comparisons) were carried out considering the total data set (24 values for each variable: 3 years, 2 treatments and 4 blocks) and for each treatment separately (12 values: 3 years and 4 blocks).

## Results

### Weed community and density

Weed community in the present study consisted of a variable number of species with a relative low density and a few dominant species, as normally occurs in farming systems. Six annual weed species: *Abutilon theophrasti* Med, *Amaranthus retroflexus* L., *Chenopodium album* L., *Echinochloa colona* (L.) Link, *Portulaca oleracea* L. and *Solanum nigrum* L.; and two perennial: *Convolvulus arvensis* L. and *Malva sylvestris* L., were recorded during the study.

Table 2 shows the density and frequency of *E*. *colona*, *P*. *oleracea*, and *S*. *nigrum*, the dominant weed species, found at least in 5% of the quadrats in one of the conducted surveys, under conventional or glyphosate herbicide regime. The other species were found to a much lesser extent: *A*. *retroflexus*, *C*. *album* and *M*. *sylvestris* were not detected in the C system and only occasionally (one sampling time) in the G system, while *A*. *theophrasti* and *C*. *arvensis* appeared occasionally in both herbicide systems. Both herbicide management regimes reduced the density of weeds, but the response of each species depended on the herbicide treatment. In plots managed with conventional herbicides, the application of the pre-emergence herbicide effectively reduced the emergence of weeds at ST1 and ST2, except for *E*. *colona* (Table 2). However, *E*. *colona* was completely controlled in 2009 and 2010 after the application of the post-emergence treatment. In plots managed with glyphosate, *E*. *colona*, *P*. *oleracea*, and *S*. *nigrum* were the most abundant weed species identified during the initial weed counts after the cotton sowing (ST1) with up to 7.94, 6.91 and 1.56 plants m^-2^, respectively (Table 2). Weed densities at ST3 show that almost all weeds except *P*. *oleracea* were suppressed by the two post-emergence glyphosate treatments. In 2008 and 2009, the first glyphosate treatment was applied when cotton plants were at 4th leaf stage, but *P*. *oleracea* plants were then at the reproductive stage and survived the effect of the herbicide. When cotton received the second glyphosate application at the 8th leaf stage, the size of *P*. *oleracea* at that moment compromised the herbicide’s efficacy: most of the plants could complete their life cycle and left a great seedbank for the following years. In 2010, glyphosate treatments were applied earlier (at 3rd and 6th leaf stage of the cotton) and most of *P*. *oleracea* plants were controlled. However, some *P*. *oleracea* plants were well developed even when cotton plants were at the cotyledon stage. Therefore, they could complete their cycle and leave seeds.

**Table 2:** Density and frequency of the main weed species (found at least in 5% of the quadrats in one of the conducted surveys) under conventional or glyphosate herbicide regime.

Weed species	Year	Weed Density^a^ (Frequency)					
		Conventional			Glyphosate		
		ST1	ST2	ST3	ST1	ST2	ST3
***Echinochloa colona***	2008	0.97 ± 0.38 (0.15 ± 0.04)	1.65 ± 0.45 (0.30 ± 0.15)	1.74 ± 0.36 (0.31 ± 0.02)	7.94 ± 2.18 (0.54 ± 0.06)	0.56 ± 0.12 (0.13 ± 0.06)	0.03 ± 0.03 (0.01± 0.01)
	2009	9.56 ± 3.39 (0.44 ± 0.06)	10.97 ± 3.50 (0.59 ± 0.07)	0	1.59 ± 0.22 (0.31 ± 0.03)	0.18 ± 0.14 (0.04 ± 0.03)	0
	2010	0.85 ± 0.35 (0.15 ± 0.06)	1.76 ± 0.38 (0.35 ± 0.08)	0	0.56 ± 0.33 (0.10 ± 0.05)	0.03 ± 0.03 (0.01 ± 0.01)	0
***Portulaca oleracea***	2008	0.06 ± 0.06 (0.01 ± 0.01)	0.06 ± 0.03 (0.01 ± 0.05)	0.06 ± 0.06 (0.01 ± 0.01)	6.91 ± 2.82 (0.65 ± 0.13)	4.06 ± 1.63 (0.56 ± 0.28)	3.44 ± 1.24 (0.49 ± 0.14)
	2009	0.03 ± 0.03 (0.01 ± 0.01)	0.06 ± 0.03 (0.01 ± 0.01)	0.03 ± 0.03 (0.01 ± 0.01)	23.50 ± 4.37 (0.92 ± 0.05)	22.88 ± 5.11 (0.87 ± 0.09)	9.53 ± 1.99 (0.84 ± 0.11)
	2010	0.03 ± 0.03 (0.01 ± 0.01)	0.09 ± 0.03 (0.04 ± 0.01)	0.03 ± 0.03 (0.01 ± 0.01)	40.62 ± 12.3 (0.94 ± 0.06)	10.82 ± 4.70 (0.67 ± 0.09)	1.12 ± 0.39 (0.19 ± 0.07)
***Solanum nigrum***	2008	0	0	0	1.56 ± 0.52 (0.27 ± 0.10)	0	0
	2009	0	0	0	0	0	0
	2010	0.06 ± 0.06 (0.01 ± 0.01)	0.09 ± 0.03 (0.02 ± 0.01)	0.12 ± 0.05 (0.03 ± 0.01)	1.26 ± 0.60 (0.24 ± 0.10)	0	0

Weed assessment was conducted in both Conventional and Glyphosate treated plots: ST1) a few days before the application of POST-G1; ST2) 2–3 weeks after POST-G1; and ST3) 2–3 weeks after POST-C/POST-G2 (see Fig 1).

^a^ Values represent the mean ± standard error (SE) of 4 plots per treatment (34 quadrats per plot).

Weed density differed significantly between both herbicide management regimes, with significant lower total weed densities in the C than in the G system (Table 3).

**Table 3:** Total density of weeds growing under conventional or glyphosate herbicide regime.

Year	Treatment	Weeds Density (mean ± SE)^a^			Year (Y) [*F*_2, 28_ (*P*)]	Treatment (T) [*F*_1, 28_ (*P*)]	Sampling (ST) [*F*_2, 28_ (*P*)]	Y x ST [*F*_4, 28_ (*P*)]	T x ST [*F*_2, 28_ (*P*)]
		ST1	ST2	ST3					
**2008**	Conventional	1.24 ± 0.60	1.62 ± 0.39	1.82 ± 0.44	28.45* (0.001)	29.46* (0.012)	64.47 (0.000)	92.30 (0.000)	62.47 (0.000)
	Glyphosate	17.0 ± 5.51	3.85 ± 1.01	3.68 ± 1.41					
**2009**	Conventional	9.59 ± 3.41	11.1 ± 3.49	0.03 ± 0.03					
	Glyphosate	25.1 ± 4.54	23.1 ± 5.20	9.53 ± 1.99					
**2010**	Conventional	1.06 ± 0.46	2.03 ± 0.56	0.24 ± 0.08					
	Glyphosate	42.7 ± 12.5	10.0 ± 3.94	0.91 ± 0.41					

Weed assessment was conducted in both Conventional and Glyphosate treated plots: ST1) a few days before the application of POST-G1; ST2) 2–3 weeks after POST-G1; and ST3) 2–3 weeks after POST-C/POST-G2 (see Fig 1). Means were compared by four-way ANOVA, and the F calculated for the factors year, treatment (herbicide regime), blocks and sampling times, as well as all associated interactions. Only significant factors and interactions are shown. Significant values (*P* < 0.05) are marked with *

^a^ Average of 4 plots per treatment (34 quadrats per plot).

Significant differences on total weed density were also detected among years, being higher in 2009 than in 2008 and 2010 (SNK, P < 0.05, Table 3). The management done in 2008, in which the late sowing of the crop resulted in the lack of post-emergence application in the C system and in a delay of glyphosate application in the G system, allowed *E*. *colona* in conventional and *P*. *oleracea* in glyphosate to escape, increasing the seeds in the soil seed bank for the next year. Sampling time and the interactions ‘treatment by sampling time’ and ‘year by sampling time’ resulted also significant in accordance the herbicide applications (Table 3). Interestingly, the ‘year by treatment’ interaction was not significant, indicating that differences between treatments were consistent over time.

### Weed species richness and diversity

Weed species richness (*S*) was relatively low across the years in both herbicide management regimes, with mean number of species ranging from 1.5 to 3.1 species per year on glyphosate management and from 1.0 to 2.1 species per year on the conventional, with no significant differences between herbicide management regimes (Table 4). There were however significant differences among years (Table 4), with the lowest richness obtained in 2009 and the highest in 2010 (SNK, P < 0.05). ‘Year by treatment’ interaction was not significant (Table 4).

**Table 4:** Species richness (S) and diversity index (H’) of weeds in glyphosate tolerant cotton under conventional or glyphosate herbicide regimes.

	Year	Treatment (mean^a^ ± SE)		Year (Y) [*F*_2, 18_ (*P*)]	Treatment (T) [*F*_1, 18_ (*P*)]	Y x T [*F*_2, 18_ (*P*)]
		Conventional	Glyphosate			
**Richness (S)**	2008	1.75 ± 0.53	3.25 ± 0.64	4.60* (0.02)	1.29 (0.27)	1.67 (0.22)
	2009	1.00 ± 0.14	1.50 ± 0.10			
	2010	2.08 ± 0.63	2.17 ± 0.10			
**Diversity (*H’*)**	2008	0.22 ± 0.15	0.56 ± 0.05	3.31 (0.06)	3.73 (0.07)	4.66* (0.02)
	2009	0.02 ± 0.01	0.09 ± 0.01			
	2010	0.43 ± 0.16	0.10 ± 0.02			

Means were compared by two-way ANOVA, and the *F* calculated for the two factors (year and treatment) and their interaction. Significant values (*P* < 0.05) are marked with *

^a^ Data were pooled across sampling dates within plots

There was a significant ‘year by treatment’ interaction after the ANOVA on weed diversity data (Table 4), showing that weed diversity (Shannon-Wiener index, *H’*) response was not consistent from 2008 to 2010, so differences between herbicide management regimes were re-analysed within each year. There were no differences in weed diversity between glyphosate- and conventionally-managed plots in the year 2008 (F_1,6_ = 5.17, *P* = 0.063), whereas in 2009 weed diversity resulted significantly higher in G than in C system (F_1,6_ = 57,09, *P* <0.001) and in 2010 resulted significantly lower in G than in C in 2010 (F_1, 6_ = 32.61, *P* = 0.002). This year weed presence was restricted to *P*. *oleracea* after the first treatment with glyphosate.

### Arthropod taxa

A total of 3141 arthropods captured by pitfall traps were assessed during the three-year study (Table 5). Of these, the most abundant groups considered generalist predators were spiders (Araneae) (38%), followed by earwigs (Dermaptera) (32%), ground beetles (Coleoptera: Carabidae) (18%), rove beetles (Coleoptera: Staphylinidae) (9%) and centipedes (Chilopoda:Lithobiidae) (3%). Other groups found were crickets (Orthoptera: Gryllidae) of the genera *Acheta* and *Gryllus* (51% of the total), which feed on vegetable material or small prey; woodlices (Isopoda) of the family Porcellionidae (4%), mostly detritivores, and darkling beetles (Coleoptera: Tenebrionidae) (3%) which feed on both fresh and decaying vegetation

**Table 5:** Total number of the ground-dwelling arthropod groups captured in pitfall traps in glyphosate tolerant cotton plots.

Arthropod group^a^	2008	2009	2010	Total
**Crickets (ORT)**	934	411	240	1585
**Spiders (ARA)**	108	161	233	502
**Earwigs (DER)**	278	91	57	426
**Ground beetles (COL)**	118	69	48	235
**Woodlice (ISO)**	41	18	91	150
**Rove beetles (COL)**	55	50	15	120
**Darkling beetles (COL)**	3	28	50	81
**Centipedes (CHI)**	2	35	5	42
**Total**	1539	863	739	3141

^a^ In brackets: ORT, Orthoptera; ARA, Araneae; DER, Dermaptera; COL, Coleoptera; ISO, Isopoda; CHI, Chilopoda.

The composition of the predatory groups varied among years. Thus, spiders’ relative activity-density ranged from 19% of the total predators in 2008 to 65% in 2010. In 2008, 50% of the total predators were earwigs, but it was only 16% in 2010. Ground beetles ranged from 21% of the total predators in 2008 to 13% in 2010, and rove beetles between 10% in 2008 and 4% in 2010 (Table 5).

### Activity-density predator patterns and community indicators

**Fig 2:**
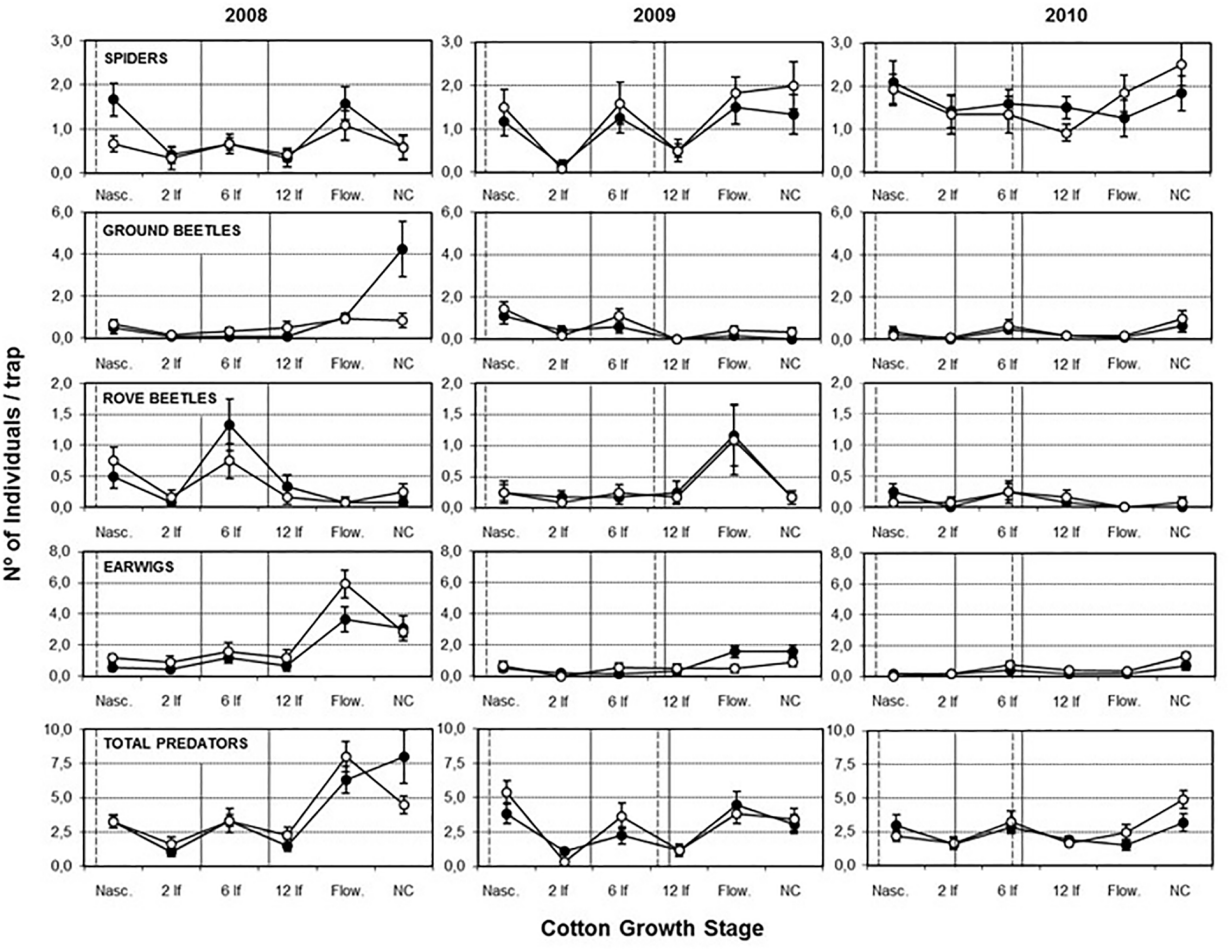
Activity-density patterns of the four main predatory groups: Spiders, ground beetles, rove beetles and earwigs. Mean number of individuals per trap (± SE) of the four most abundant ground-dwelling predator groups in glyphosate tolerant cotton under glyphosate (○) or conventional herbicides (●) regime. Sampling dates on the *x*-axis are cotton nascence (Nasc.), cotton in 2nd (2 lf), 6th (6 lf), and 12th (12 lf) leaf stage, cotton flowering (Flow.) and after cotton extraction (NC -No Crop-). The vertical lines indicate the glyphosate (—) and the conventional (- -) treatments.

The activity-density patterns of the four main predatory groups (spiders, ground beetles, rove beetles and earwigs) oscillated throughout the cotton phenological stage (Fig 2). Only in the case of ground beetles a major peak was recorded in plots treated with conventional herbicides in the last sampling date of 2008 (with the crop already extracted), but not in the following years.

**Fig 3:**
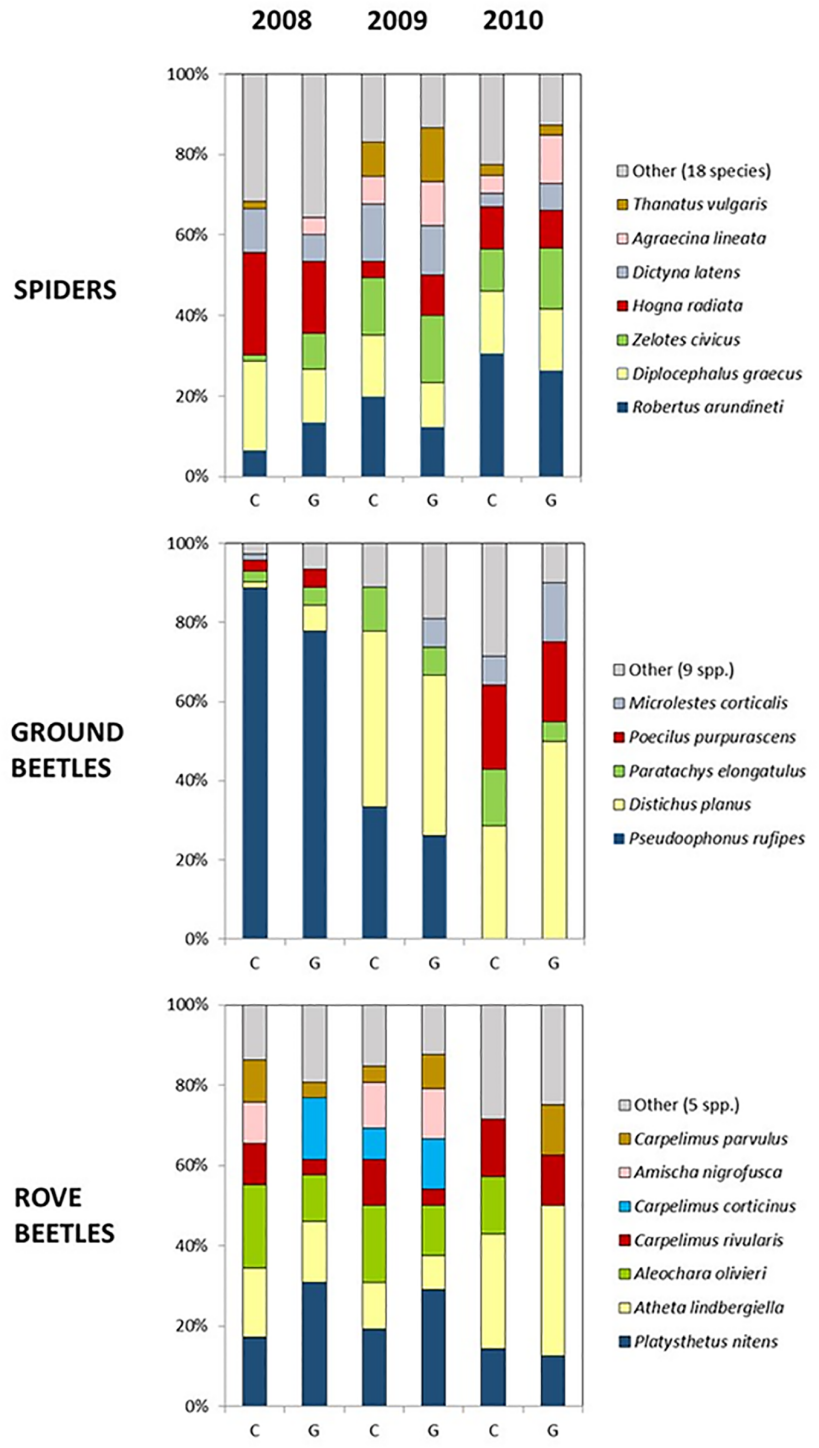
Species composition of spiders, ground beetles and rove beetles in glyphosate tolerant cotton under conventional (C) or glyphosate (G) herbicide regime. Other species: Ground Beetles (Carabidae): *Siagona europea*, *Calosoma maderae*, *Sphaerotachys lucasii*, *Scybalicus oblongiusculus*, *Paratachys bistriatus*, *Zuphium olens*, *Microlestes sp*., *Bembidion vicinum*, *Ophonus ardosiacus*; Rove beetles (Staphylinidae): *Xantholinus vandalicus*, *Scopaeus mitratus*, *Achenium depressum*, *Anotylus nitidulus*, *Atheta laticollis*; Spiders (Araneae): Pardosa sp. (Lycosidae), Aelurillus sp. (Salticidae), Agroeca sp. (Liocranidae) and other 15 spider morphospecies that were not identified because of their low abundance (less than 10 captures in total).

The differences found in the activity-density values for these taxonomic groups strongly depended on the year, but no differences were observed between treatments (Table 6). The ‘year by treatment’ interaction was only significant for earwigs. Twenty-five species of spiders were found during the three years of sampling. Seven of them accounted for 80% of the total: *Robertus arundineti* O.P.-Cambridge (Theridiidae), *Diplocephalus graecus* O.P.-Cambridge (Linyphiidae), *Zelotes civicus* Simon (Gnaphosidae), *Hogna radiata* Latreille (Lycosidae), *Dictyna latens* Fabricius (Dictynidae), *Agraecina lineata* Simon (Liocranidae) and *Thanatus vulgaris* Simon (Philodromidae) (Fig 3). Significant changes among years were recorded for the activity-density of *R*. *arundineti* (*F*_2, 48_ = 25.94, *P* < 0.001), *Z*. *civicus* (*F*_2, 48_ = 11.77, *P* < 0.001), *T*. *vulgaris* (*F*_2, 48_ = 9.08, *P* < 0.001), *A*. *lineata* (*F*_2, 48_ = 4.61, *P* = 0.015) and *D*. *graecus* (*F*_2, 48_ = 3.54, *P* = 0.037). However, only *H*. *radiata* showed significant differences between treatments, the activity-density being higher under the glyphosate herbicide regime (*F*_1, 48_ = 7.92, *P* = 0.007), and *A*. *lineata* for ‘year by treatment’ interaction (*F*_2, 48_ = 3.40, *P* = 0.042).

**Table 6:** Activity-density of ground-dwelling predatory groups in glyphosate tolerant cotton under conventional or glyphosate herbicide regimes.

Group	Year	Treatment (mean^a^ ± SE)		Year (Y) [*F*_2, 48_ (*P*)]	Treatment (T) [*F*_1, 48_ (*P*)]	Y x T [*F*_2, 48_ (*P*)]
		Conventional	Glyphosate			
**Spiders**	2008	0.88 ± 0.13	0.63 ± 0.10	29.09* (0.00)	0.00 (0.95)	2.43 (0.10)
	2009	0.99 ± 0.14	1.25 ± 0.17			
	2010	1.61 ± 0.16	1.64 ± 0.17			
**Ground beetles**	2008	1.00 ± 0.28	0.57 ± 0.10	10.33* (0.00)	0.47 (0.49)	3.14 (0.05)
	2009	0.38 ± 0.10	0.57 ± 0.11			
	2010	0.29 ± 0.08	0.38 ± 0.09			
**Rove beetles**	2008	0.40 ± 0.10	0.36 ± 0.07	15.16* (0.00)	0.35 (0.56)	0.18 (0.83)
	2009	0.36 ± 0.10	0.33 ± 0.11			
	2010	0.10 ± 0.04	0.11 ± 0.04			
**Earwigs**	2008	1.60 ± 0.26	2.26 ± 0.30	70.87* (0.00)	3.63 (0.06)	4.82* (0.01)
	2009	0.74 ± 0.13	0.53 ± 0.11			
	2010	0.29 ± 0.06	0.50 ± 0.09			

Means were compared by a mixed-model ANOVA (year and treatment as fixed factors and block as random) with repeated measures (sampling date) analyses. All factors’ interactions were studied but only ‘year by treatment’ values are shown. Significant values (*P* < 0.05) are marked with *

^a^ Means per pitfall trap

Both spider’s richness and diversity did not significantly differ between treatments and among years (Table 7). A total of fourteen species of ground beetles were collected in pitfall traps in the three-year field study. Five species accounted for 84% of the total number of individuals collected: *Pseudoophonus rufipes* DeGeer, *Distichus planus* Bonelli, *Paratachys elongatulus* Dejean, *Poecilus purpurascens* Dejean and *Microlestes corticalis* Dufour (Fig 3). There were significant differences in the activity-density of *D*. *planus* depending on the year (*F*_2, 48_ = 12.04, *P* < 0.001), treatment (*F*_1, 48_ = 15.44, *P* < 0.001) and ‘year by treatment’ interaction (*F*_2, 48_ = 9.62, *P* < 0.001). Likewise, significant changes were recorded for *P*. *rufipes* among years (*F*_2, 48_ = 48.81, *P* < 0.001) and between treatments (*F*_1, 48_ = 5.83, *P* < 0.001), and for *P*. *purpurascens* between treatments (*F*_1,48_ = 6.37, *P* < 0.015). Richness values for ground beetles were significantly affected by treatment, but not by year, whereas diversity values significantly differ between treatments and among years (Table 7). In those cases where treatment was a significant factor, the estimated parameter was higher under the glyphosate herbicide regime

**Table 7:** Species richness (S) and diversity index H’) of spiders, ground beetles and rove beetles in glyphosate tolerant cotton under conventional or glyphosate herbicide regimes.

	Year	Treatment (mean^a^ ± SE)		Year (Y) [*F*_2, 18_ (*P*)]	Treatment (T) [*F*_1, 18_ (*P*)]	Y x T [*F*_2, 18_ (*P*)]
		Conventional	Glyphosate			
**Richness (*S*)**						
Spiders	2008	7.0 ± 0.7	7.0 ± 2.0	3.23 (0.06)	0.01 (0.94)	0.22 (0.81)
	2009	9.0 ± 1.9	9.8 ± 1.4			
	2010	10.8 ± 0.5	9.8 ± 0.8			
Ground beetles	2008	3.0 ± 1.2	3.5 ± 0.5	0.21 (0.81)	4.64* (0.04)	0.79 (0.47)
	2009	2.5 ± 0.5	5.0 ± 0.6			
	2010	3.0 ± 0.9	4.3 ± 0.9			
Rove beetles	2008	5.3 ± 0.3	4.3 ± 0.9	5.15* (0.02)	0.25 (0.62)	0.13 (0.88)
	2009	4.5 ± 1.7	4.3 ± 1.3			
	2010	1.8 ± 0.5	1.8 ± 0.8			
**Diversity (*H’*)**						
Spiders	2008	1.76 ± 0.12	1.69 ± 0.27	2.34 (0.13)	0.23 (0.88)	0.12 (0.89)
	2009	1.98 ± 0.16	2.04 ± 0.12			
	2010	2.02 ± 0.03	1.97 ± 0.05			
Ground beetles	2008	0.35 ± 0.21	0.77 ± 0.19	3.69* (0.04)	9.91* (0.01)	0.27 (0.76)
	2009	0.69 ± 0.15	1.39 ± 0.10			
	2010	0.81 ± 0.31	1.26 ± 0.21			
Rove beetles	2008	1.61 ± 0.03	1.29 ± 0.22	7.61* (0.00)	0.27 (0.61)	0.24 (0.79)
	2009	1.17 ± 0.44	1.24 ± 0.24			
	2010	0.45 ± 0.27	0.33 ± 0.33			

Means were compared by two-way ANOVA, and the *F* calculated for the two factors (year and treatment) and their interaction. Significant values (*P* < 0.05) are marked with *

^a^ Data were pooled across sampling dates within plots

Twelve different species of rove beetles were recorded in the pitfall traps along the three-year field survey. Among them, seven species account for 83% of the total: *Platysthetus nitens* Sahlberg, *Atheta lindbergiella* Brundin, *Aleochara olivieri* Fauvel, *Carpelimus rivularis* Motschulsky, *Carpelimus corticinus* Gravenhorst, *Amischa nigrofusca* Stephens and *Carpelimus parvulus* Mulsant et Rey (Fig 3). No significant differences in activity-density were detected between the two treatments in any of these species, whereas significant changes among years were found for *A*. *olivieri* (*F*_2, 48_ = 4.07, *P* = 0.023), *P*. *nitens* (*F*_2, 48_ = 4.40, *P* = 0.018) and *A*. *nigrofusca* (*F*_2, 48_ = 3.45, *P* = 0.040). Richness and diversity values were significantly lower in 2010 than in the other two sampling years because of the limited catches of rove beetles in pitfall traps, but no significant differences were found between treatments (Table 7).

Only three species of earwigs were collected during the three-year field survey: *Labidura riparia* (57%), *Nala* sp. (40%) (Dermaptera: Labiduriidae) and *Labia* sp. (Dermaptera: Labiidae) (3%). Therefore, richness (*S*) and diversity indices were not calculated. No significant differences in activity-density were detected for any of these species between the two treatments, and only *L*. *riparia* showed significant changes among years (*F*_2, 48_ = 121.15, *P* < 0.001).

No correlation was shown between the activity-density of the main predatory groups and the density of weeds, when both treatments (glyphosate and conventional) were considered in the Pearson’s correlation analysis. However, a significantly positive correlation was recorded between total weed density and ground beetle richness and diversity (*r* = 0.41, *P* = 0.04 and *r* = 0.54, *P* < 0.01, respectively). When considering each treatment separately, weed density was positively correlated to spiders’ activity-density, richness and diversity (*r* = 0.73, *P* < 0.01; *r* = 0.73, *P* < 0.01 and *r* = 0.60, *P* = 0.04, respectively) and to carabids’ diversity (*r* = 0.68, *P* = 0.02) in plots treated with glyphosate.

## Discussion

Cotton production is especially threatened by weed competition during the early stages of development [39]. Conventional pre-emergence chemical control reduces or delays weed growth, with few dominant weed species and to a relatively low densities, to such an extent that the cotton plants can successfully compete with the later developing weeds. The cultivation of GMHT crops entails the use of more flexible weed management systems that offer the opportunity to delay control until weeds emerge. However, it might cause changes in the composition and abundance of weed species present in the cropping systems. This 3-year field-scale study revealed that glyphosate-only treatments (two applications in post-emergence) for weed control in GT cotton for three consecutive years favoured changes of the floristic composition, which led to an increase in the abundance of *P*. *oleracea* in glyphosate-treated plots at the initial pre-emergence samplings (ST1) from year to year. These findings can be associated to the fact that only young *P*. *oleracea* plants are completely controlled by glyphosate [40,41]. Besides, some biological traits of *P*. *oleracea*, such as short ripening time (39 to 57 days after emergence), high reproductive potential (more than a quarter of million seeds per season), several seed generations per season, and seeds viability in soils for up to 30 years, facilitates its rapid response to management changes [42,43]. Thus, the timing on the application of glyphosate is therefore essential in terms of weed control efficacy. The application of glyphosate when the cotton was at the 2–3 and 6–7 leaf stages gave exceptional control of *P*. *oleracea*, as shown in 2010, but the later emergence of other weeds could require subsequent applications or mechanical weeding. On the other hand, if weeds are not treated timely, as happened in 2008 and 2009, most of them are eradicated, but the control for *P*. *oleracea* is less efficient. In addition, drought during 2009 (rainfall between May and August was 8.8 mm in comparison with the average of 58 mm in the last 30 years) [44] could have contributed to reduce herbicide effectiveness in the glyphosate-treated plots, as post-emergence herbicides are generally less effective for controlling weeds that are subjected to high temperature or drought. These stresses generally reduce absorption, translocation and metabolism of herbicides [45]. Thus, the seed bank was replenished with *P*. *oleracea* seeds for the next years, and an increase in the active ingredient rate or in the treatment frequency would be required to achieve an efficient control [46].

Therefore, our results suggest that *P*. *oleracea* is a likely candidate for weed shifts in GT cotton, as reported in continuous glyphosate-treated GT soybean and maize [47]. Glyphosate-induced weed shifts have already been documented in commercial fields in the USA and Australia [33,48,49], for weeds that are less susceptible to glyphosate, as for example, *Ipomoea* spp., *Commelina* spp., *Cyperus* spp., *Abutilon theophrastii* Med. or *Ambrosia trifida* L. [50–51], and weeds that escape control due to their biological characteristics, such as *C*. *album* L., *Digitaria sanguinalis* (L.) Scop., *Setaria* spp. or *Echinochloa* spp., [52–55].

A major species shift towards *Conyza bonariensis* was observed in a glyphosate-based system in Australian cotton [49]. Then, the use of additional pre-emergence herbicides to achieve good weed control, to reduce selection pressure, and to avoid a possible weed shift may be imposed. In fact, several combinations of glyphosate with pre-emergence residual herbicides have been already recommended for a better control of recalcitrant weeds [55]. However, this practice will equate the management done in both glyphosate and conventional systems.

Post-emergence weed control strategies in GT crops are also important in terms of their effects on weed flora diversity [56]. As such, we did observe a changing scenario in weed diversity throughout the three years. While in 2008 and 2009 no significant differences were observed in weed diversity between herbicide management regimes, in the last year diversity was higher under the conventional herbicide management. The observed changes after three years were probably due to the different herbicide use: a residual herbicide in pre-emergence that allows the presence of *E*. *colona* until the application of a post-emergence graminicide; and a control with post-emergence treatments with glyphosate, leading to a shift in weed species composition, where *P*. *oleracea* became the almost only weed present in the field.

Changes in the weed community have been reported to cause alterations in the arthropod fauna [6,10]. In our study, no significant differences between both herbicide regimes were observed in the activity-density of the main groups of ground-dwelling predators captured. Similar results were obtained when the most abundant species within each group were analyzed, with the only exception of three species of carabids (*D*. *planus*, *P*. *rufipes* and *P*. *purpurascens*) and one of spiders (*H*. *radiata*) that were more abundant in glyphosate treated cotton. However, significant differences between treatments were obtained for ground beetles species richness and diversity and a positive correlation with weed density could be established for both parameters. These result are in agreement with other field studies, where it has been reported that higher weed biomass can sustain a greater number of prey species, which then attract more predators [22,26–28]. Since total weed density was higher under glyphosate herbicide regime, ground beetles can have a wider target spectrum in these plots than in the conventional treated ones. In addition, the shift in favour to *P*. *oleracea* in plots managed with glyphosate, could also be related with the positive correlation between weed density and the diversity of carabids in these plots. This effect can be particularly relevant for those species that have both carnivorous and granivorous feeding habits, which can take advantage of the higher availability of weed seeds late in the season under GMHT treatment. Indeed, it has been reported that the changes in weed composition, density, and seed availability can affect the activity of some carabid species in the field [57,58].

Differences in the activity-density of all predatory groups were observed among years, with a tendency to a significant reduction in ground beetles, rove beetles and earwigs and a significant increase of spiders through the three years of the study in both conventional and glyphosate herbicide regimes. Similarly, significant differences for rove beetles richness and diversity and ground beetles diversity were also obtained among years, which in the case of rove beetles also showed a reduction through the three years of the study. As in this field survey, other field studies have also revealed negative relationship between spiders, carabids and rove beetles, suggesting that interspecific competition may occur between these groups [31,59]. This matter was especially relevant in the glyphosate-treated plots, dominated by *P*. *oleracea* plants, where strong positive correlations between weed density and spiders’ activity-density, richness and diversity were detected. It is known that spiders are very sensitive to changes in the habitat structure, particularly when modifications are made to the vegetation structure and species composition [60,61]. The anatomical structure of *P*. *oleracea*, being a prostrate plant covering an expanded soil surface, could have helped spiders to settle in the field outcompeting other predators. In addition, intraguild predation of other generalist predators by spiders has been reported in cotton [62,63], which may have also contributed to reshaping predator community composition.

## Conclusions

Our findings suggest that weed control strategies with exclusive reliance on repeated glyphosate applications can reduce weed diversity and induce a shift in weed species composition in GT cotton. No significant differences were observed in the activity-density of ground-dwelling predators between conventional and glyphosate herbicide regimes, though an increase in ground beetles species richness and diversity was observed in glyphosate treated cotton. Yet, to prevent a reduction in weed diversity while maintaining some weed abundance it is crucial to avoid using the same herbicide year after year and to rotate herbicides and crops.

## Supporting information

S1 Fig Agro-ecosystem in which the field assay were conducted and experimental design. A) Aerial view of the 2 ha field assay sown with GMHT cotton at Lebrija from 2008–2010. B) Experimental design: the trial was laid out as a randomized complete block design, with two weed-management regimes and four blocks of two plots. The two treatments were randomly assigned to the two plots within each block and maintained for three years. Plot size was 60 m by 30 m. C: treatment with herbicide used conventionally in cotton. G: treatment with glyphosate. (+) pitfall traps used to monitor aboveground arthropods, with three traps arranged diagonally across each plot. (o) quadrats used for weed assessment, with thirty-four quadrats per plot placed in a zig-zag pattern along two diagonal transects (ten quadrats per transect, only one diagonal is shown) and in the four borders (three quadrats per short border and four per long border).
(TIF)
