# Supplementary figures and images for: Weeds and ground-dwelling predators′ response to two different weed management systems in glyphosate-tolerant cotton: A farm-scale study
Source: PLoS One. 2018 Jan 19;13(1):e0191408. doi: 10.1371/journal.pone.0191408 (PMC5774765; doi:10.1371/journal.pone.0191408)

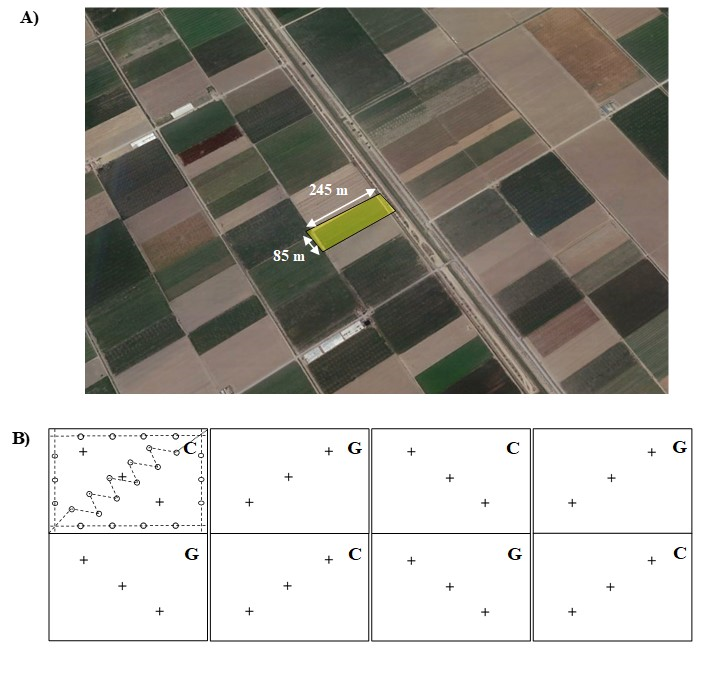

Supplement: S1 Fig — A) Aerial view of the 2 ha field assay sown with GMHT cotton at Lebrija from 2008–2010. B) Experimental design: the trial was laid out as a randomized complete block design, with two weed-management regimes and four blocks of two plots. The two treatments were randomly assigned to the two plots within each block and maintained for three years. Plot size was 60 m by 30 m. C: treatment with herbicide used conventionally in cotton. G: treatment with glyphosate. (+) pitfall traps used to monitor aboveground arthropods, with three traps arranged diagonally across each plot. (o) quadrats used for weed assessment, with thirty-four quadrats per plot placed in a zig-zag pattern along two diagonal transects (ten quadrats per transect, only one diagonal is shown) and in the four borders (three quadrats per short border and four per long border). (TIF) [file pone.0191408.s001.tif]
